# Assessing the Importance of Pyrolysis Process Conditions and Feedstock Type on the Combustion Performance of Agricultural-Residue-Derived Chars

**DOI:** 10.1021/acs.energyfuels.0c04180

**Published:** 2021-02-03

**Authors:** Joan J. Manyà, Darío Alvira, María Videgain, Gozde Duman, Jale Yanik

**Affiliations:** † Aragón Institute of Engineering Research (I3A), Thermochemical Processes Group, University of Zaragoza, Escuela Politécnica Superior, crta. Cuarte s/n, 22071 Huesca, Spain; ‡ University of Zaragoza, Escuela Politécnica Superior, crta. Cuarte s/n, 22071 Huesca, Spain; § Faculty of Science, Department of Chemistry, Ege University, 35100 Bornova, Izmir, Turkey

## Abstract

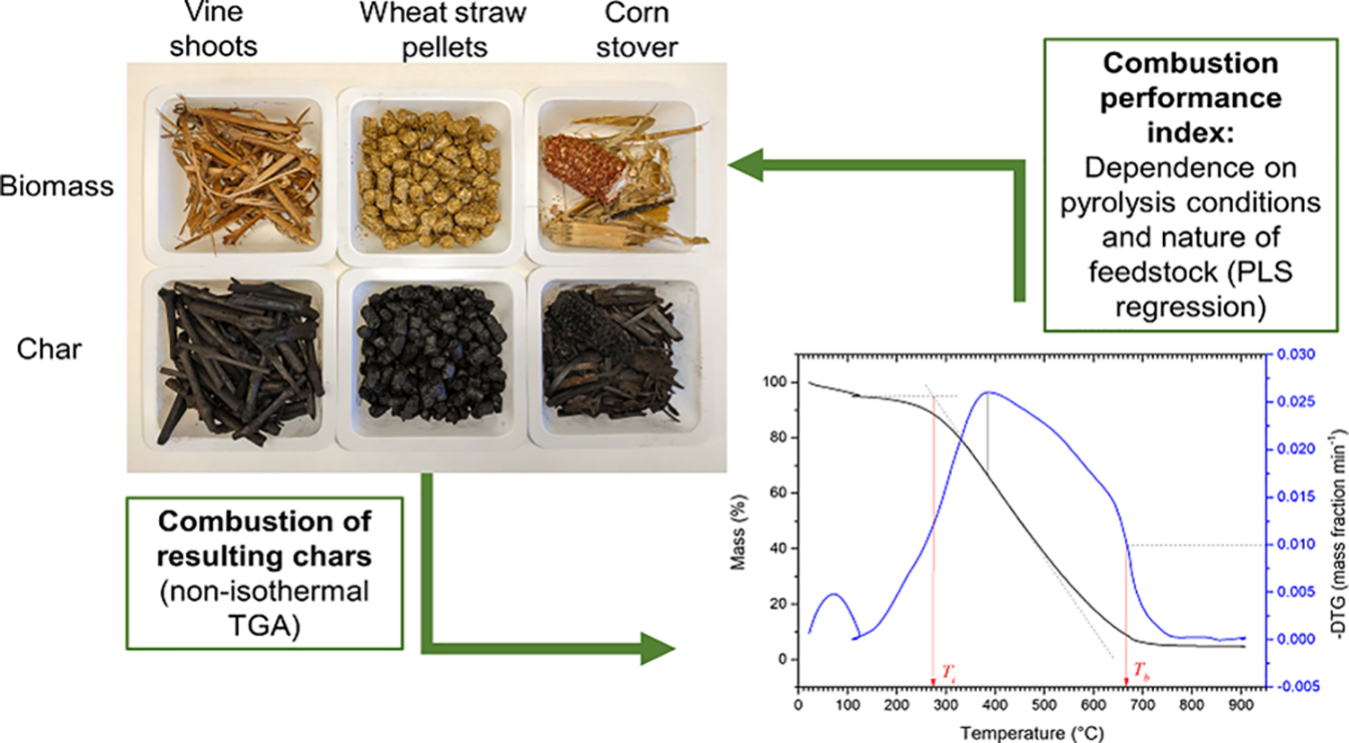

The combustion performance of chars derived from vine shoots, wheat straw, and corn stover was investigated to assess the influence of both the biomass precursor and pyrolysis operating conditions. Chars were produced through slow pyrolysis at different peak temperatures (350 and 500 °C), pressures (0.1 and 0.5 MPa), and residence times of the vapor phase (50 and 150 s). From the thermogravimetric curves obtained under air, the combustion performance index (*S*) was calculated for each char. Apparent kinetics were also estimated using the Coats–Redfern method and assuming an F3/2 reaction model. Results show that the combustion patterns of chars were more influenced by the type of feedstock than by the pyrolysis conditions. Corn stover appeared to be the most interesting feedstock in order to produce chars with tuned reactivity. Results from partial least-squares (PLS) regression revealed that the most important factors affecting *S* were the contents of potassium (negative effect) and cellulose (positive effect) in the original biomass.

## 1 Introduction

Biomass is the only renewable resource of carbonaceous fuels, and therefore, it has attracted considerable attention as a replacement for coal in both power plants and domestic heating. Nevertheless, if directly used as fuel, biomass features some drawbacks, such as a low energy density, low calorific value, and high energy requirement for grinding. Thermochemical conversion technologies (such as slow pyrolysis, torrefaction, and hydrothermal carbonization) are valuable pathways for converting lignocellulose biomass into a char product with improved fuel quality.^1^

Slow pyrolysis is a well-known process, in which biomass is slowly heated under an inert gas environment up to typically 350–650 °C. A lot of research has been done on the effects of pyrolysis operating conditions and biomass feedstock on the yield and physicochemical properties of produced chars.^2−6^ However, more efforts are required to better clarify the above-mentioned effects on the performance of biomass char as an energy carrier.

To assess the fuel properties of biomass char, both the ignition and burnout temperatures are commonly used to describe the combustion behavior.^7^ The ignition temperature is defined as the minimum temperature at which a given fuel ignites spontaneously in an environment without any external source of ignition.^8^ For its part, the burnout temperature refers to the temperature at which the fuel is almost completely consumed.^7^ From thermogravimetric analysis (TGA) data, it is possible to estimate both ignition and burnout temperatures and also calculate the so-called combustion performance index (*S*), as has recently been reported by Mundike et al.^9^ and Wang et al.^10^ This index is a measure of the burning ability of a fuel, and a higher value reflects a more satisfactory combustion performance.^11^ Knowledge of the combustion performance and kinetics of biomass chars is of great importance to properly design industrial-scale combustors, where the residence time of fuel particles is very short.^12^

Previous research has compared the combustion (or cocombustion) performance of a number of biomass-derived chars produced from different feedstocks and under different operating conditions.^13−16^ These preliminary studies pointed out that char reactivity is affected by pyrolysis operating conditions under which char is obtained. The most assessed parameter was the pyrolysis peak temperature, for which an inversely proportional relationship with char reactivity has been reported.^13,14,16^ Regarding the effect of pyrolysis pressure, Recari et al.^16^ reported a gradual decrease in the reactivity of spruce-derived chars when pyrolysis was conducted at 1.0 and 2.0 MPa. The authors attributed this finding to the promotion of secondary charring reactions during pressurized pyrolysis. Furthermore, char combustion reactivity is strongly affected by the nature of the biomass feedstock. In this sense, the availability of alkaline and alkaline-Earth species in the ash can catalyze the reaction of combustion.^14^ Recently, Pang et al.^15^ reported that lignocellulosic composition of raw biomass plays a key role in determining the morphology and reactivity of the resulting chars. The authors stated that biomass with relatively high contents of lignin and cellulose could lead to increased amounts of low reactive thick-walled chars. In a more recent study, Yan et al.^17^ confirmed the negative effect of the lignin content on the reactivity of biomass-derived chars.

Agricultural residues from crops have a great potential as renewable energy source, given their truly sustainable availability, which was estimated at 85 millions of tons per year in the EU.^18^ A significant fraction of them comes from maize, wheat, and vine crops in the form of corn stover, wheat straw, and vine shoots (from pruning), respectively. Hence, in-depth studies on the combustion characteristics of chars produced from these precursors through slow pyrolysis at different operating conditions are highly encouraged.

For all the reasons mentioned above, the present study aims to investigate the effects of both the biomass precursor and pyrolysis operating conditions on the combustion performance and relative reactivity of the resulting chars. As operating pyrolysis process parameters, the following was considered in our study: peak temperature (350 and 500 °C), absolute pressure (0.1 and 0.5 MPa), and residence time of the gas phase within the pyrolysis reactor (50 and 150 s). The combustion behavior of all produced chars was investigated using thermogravimetric analysis (TGA) under dynamic (i.e., nonisothermal) heating conditions.

## 2 Experimental Section

### 2.1 Materials

Three biomass precursors were selected in this study: vine shoots (VS), wheat straw (WS), and corn stover (CS). Vine shoots (*Vitis vinifera L*.) of the grape variety Cabernet Sauvignon were collected during winter pruning in a vineyard located in the wine region of Somontano (Huesca province, Spain). They were selected by diameter (between 8.5 and 15 mm) and cut into smaller pieces of 4–7 cm in length. Wheat straw (*Triticum spp*.) pellets (7 mm OD and approximately 12 mm long) were supplied by a Belgian company, and no binder was used in making them. Corn stover (*Zea mays*), which was collected after cob harvesting in an irrigated field located in the province of Huesca (Spain), consisted of a mixture of corncob (15.4 wt %), leaf (80.1 wt %), and stalk (4.5 wt %). Leaves were cut into pieces of 14–16 cm in length and 1.0–2.0 mm in thickness. Relatively large particle sizes were used for two reasons: (1) to improve the carbonization efficiency (i.e., fixed-carbon yield) during pyrolysis, since using larger particles promotes the secondary charring reactions at an intraparticle level;^19^ and (2) to avoid high-energy-intense biomass pretreatments for size reduction.

For all the biomass sources, proximate analyses were performed according to the procedure described below. Briefly, 1 g of powdered sample was weighed on a predried ceramic crucible and placed in a convection oven at 105 ± 5 °C for at least 4 h. After weighing, the sample was placed back into the oven at the same temperature until a constant dry weight was reached. To estimate the volatile matter content, the crucible containing the resulting oven-dried sample (with the lid placed on) was put in a muffle furnace at 925 ± 10 °C for 7 min. Finally, the ash content was determined by putting the open crucible containing the resulting volatile-free sample in the muffle furnace at 730 ± 10 °C for at least 2 h.

A CHN628 elemental analyzer from LECO (USA) was used to conduct the ultimate analyses in accordance with the ASTM Standard D5373-16. In addition, X-ray fluorescence (XRF) spectroscopy analysis (using an ADVANT’XP+XRF spectrometer from Thermo ARL, Switzerland) was performed in order to determine the inorganic constituents of the biomass ash according to ASTM Standard D4326-04.

Neutral detergent fiber (NDF), acid detergent fiber (ADF), and acid detergent lignin (ADL) were determined for all the biomass sources using a fiber analyzer (ANKOM 200, USA) and according to the method proposed by Van Soest et al.^20^ Thus, it was possible to estimate the lignocellulosic constituents from the above-mentioned parameters as follows:^21^ lignin (ADL), cellulose (ADF – ADL), and hemicelluloses (NDF – ADF). Organic extractives were previously extracted in a dried cotton cellulose thimble, which was inserted in a Soxhlet extractor, using a mixture of ethanol and toluene (1:2 v/v) as solvent.

### 2.2 Production and Characterization of Chars

Chars from the three biomass sources (at the particle size ranges detailed above) were produced through slow pyrolysis at the above-mentioned different operating conditions. Biomass feedstock was heated at an average heating rate of 5 °C min^–1^ to the desired peak temperature and then held for a soaking time of 60 min (at that temperature) to ensure the thermal equilibrium. The initial sample mass of biomass was approximately 300 g for WS and VS and 130 g for CS, due to its lower apparent density.

The bench-scale pyrolysis device consisted of a cylindrical and vertical reactor (140 mm ID and 465 mm long) made of EN 1.4835 austenitic chromium–nickel steel. The corresponding schematic diagram is shown in Figure S1 (Supporting Information). More details regarding the configuration of the system are available in previous publications.^22,23^ A back-pressure regulator was used to maintain the pressure of the pyrolysis reactor at a desired value. The volumetric flow rate at standard temperature and pressure (STP) conditions of the carrier gas (N_2_) was adjusted to keep a constant real flow rate of N_2_ within the reactor (at the corresponding pressure and peak temperature) of 6.48 or 2.09 L min^–1^ to get carrier gas residence times of 50 and 150 s, respectively.

Produced chars were characterized by both proximate and ultimate analyses following the same procedures as described in Section 2.1. Results from these analyses were used to determine the fixed-carbon content (*x*_FC_) and atomic H:C and O:C ratios. The high heating value (HHV) of solid fuels (for both biomass sources and derived chars) was measured using a calorimeter (model C-200) from IKA (Germany).

Due to the highly microporous structure of biomass-derived pyrolysis chars,^24^ specific surface areas (*S*_BET_) were determined from the CO_2_ adsorption isotherms at 0 °C (using an ASAP 2020 gas sorption analyzer from Micromeritics, USA). Samples (120–175 mg) were previously degassed under dynamic vacuum conditions to constant weight at 150 °C. Pore size distribution and ultramicropore volume (*V*_ultra_) were estimated using a density functional theory (DFT) model for slit-pore geometry.

### 2.3 Combustion Behavior of Chars

Thermogravimetric curves under air atmosphere were obtained for all the chars using a TGA device (model STA 449 F3 Jupiter system) from Netzsch (Germany). Approximately 100 mg of char, which was previously crushed and sieved to a fraction of 150–500 μm, was first heated under N_2_ (100 mL min^–1^ STP) from room temperature to 110 °C (with a soaking time of 30 min) to ensure complete drying. Then, the atmosphere was switched to air (100 mL min^–1^ STP), and dried samples were heated at 10 °C min^–1^ up to 900 °C. Raw TGA curves were corrected by the corresponding blank test.

From experimental TG and DTG curves, the following parameters were determined: ignition temperature (*T*_i_), burnout temperature (*T*_b_), temperature at which the highest mass-loss rate took place (*T*_max_), and combustion performance index (*S*). *T*_i_ was estimated according to the intersection method (IM),^7^ whereas *T*_b_ was identified at the temperature where the combustion rate diminished to less than 1 wt % min^–1^.^9^*S* was calculated according to eq 1, where *DTG*_max_ and *DTG*_mean_ correspond to the maximum (at *T*_max_) and mean values (between *T*_i_ and *T*_b_) of the DTG curve, respectively.^9,11^
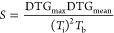
1

### 2.4 Apparent Kinetics

The Coats–Redfern (CR) procedure, which is one of several integral methods used to estimate apparent reactivity parameters from nonisothermal reaction data,^25,26^ was adopted in the present study. The apparent reaction rate of a solid–gas reaction can be expressed as follows
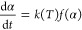
2where α corresponds to the extent of conversion (the mass loss at a given time divided by the total mass loss), *k*(*T*) is the temperature-dependent reaction rate constant, and *f*(α) is the model describing the mechanism. The Arrhenius equation, given in eq 3, is often used to describe *k*(*T*).
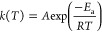
3In eq 3, *A* is the apparent pre-exponential factor, and *E*_a_ is the apparent activation energy. The expression for *g*(α), which corresponds to the integrated form of *f*(α), is obtained by rearranging eqs 2 and 3, and then integrating, leading to the following general expression
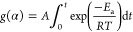
4For a constant heating rate (β = d*T*/d*t*), eq 4 can be rewritten as
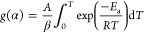
5Despite the assumption that both *A* and *E*_a_ are constant across the temperature range, the so-called temperature integral shown in eq 5 cannot be solved analytically. The CR procedure is based on a numerical approximation to the solution of the temperature integral, which results in the following linear expression^27^
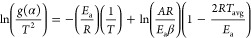
6where *T*_avg_ is the average temperature for the selected conversion range (typically 0.1–0.9). Plots of ln[*g*(α)/*T*^2^] versus 1/*T* (i.e., CR plots) will then result in straight lines, for which the slope and intercept allow an estimation of *E*_a_ and *A*, respectively.

## 3 Results and Discussion

### 3.1 Composition of Biomass Precursors

Table 1 reports the results obtained for the three feedstocks from proximate, ultimate, ash composition (as weight percentages of major oxides), and biomass constituent analyses. As the table shows, VS had a considerably higher fixed-carbon content than that of CS and WS. This fact is consistent with the higher lignin content also reported in Table 1 for VS, since lignin is the biomass constituent that gives the highest char yield.^28,29^

**Table 1:** Results from Proximate, Ultimate, Ash Composition, and Biomass Constituents Analyses of Biomass Feedstocks (VS, WS, and CS)

proximate (wt % from triplicate)	VS	WS	CS
moisture	7.97 ± 0.68	6.60 ± 0.20	7.27 ± 0.31
ash (dry basis)	1.08 ± 0.05	3.93 ± 0.28	2.70 ± 0.20
volatile matter (dry basis)	74.0 ± 1.19	83.2 ± 0.55	86.6 ± 0.11
fixed carbon (dry basis)	24.9 ± 1.91	12.8 ± 0.45	10.7 ± 0.49
ultimate (wt % in daf^a^ basis from triplicate)			
C	47.1 ± 0.14	49.0 ± 0.52	44.4 ± 0.31
H	5.29 ± 0.09	7.01 ± 0.04	5.60 ± 0.04
N	0.66 ± 0.05	0.70 ± 0.01	0.43 ± 0.01
O^b^	47.0	43.3	49.6
O:C (atomic ratio)	0.748	0.663	0.837
H:C (atomic ratio)	1.348	1.717	1.514
fuel rate^c^	0.213	0.161	0.103
HHV (MJ kg^–1^ dry basis)	18.0	17.9	18.2
lignocellulosic constituents and extractives (wt % in dry basis from duplicate)			
hemicelluloses	9.26 ± 0.97	26.9 ± 2.2	21.4 ± 0.5
cellulose	29.3 ± 1.9	37.1 ± 3.4	40.5 ± 0.9
lignin	19.2 ± 1.4	10.9 ± 1.8	9.68 ± 0.50
extractives	4.54 ± 0.37	6.57 ± 0.52	8.94 ± 0.77
inorganic matter as equivalent oxides (wt % of ash from triplicate)			
CaO	58.3 ± 0.25	25.01 ± 0.42	30.7 ± 0.23
K_2_O	18.4 ± 0.12	38.2 ± 0.45	9.85 ± 0.15
MgO	6.66 ± 0.14	2.09 ± 0.05	3.45 ± 0.17
SiO_2_	5.73 ± 0.08	24.3 ± 0.48	31.4 ± 0.23
Fe_2_O_3_	3.51 ± 0.11	0.82 ± 0.04	6.49 ± 0.12
P_2_O_5_	1.24 ± 0.06	3.20 ± 0.08	4.13 ± 0.10
Al_2_O_3_	2.57 ± 0.07	1.19 ± 0.04	4.85 ± 0.12
PbO	1.24 ± 0.04	0.32 ± 0.02	4.13 ± 0.10
S (inorganic)	0.26 ± 0.02	1.88 ± 0.05	2.50 ± 0.08
Cl (inorganic)	0.48 ± 0.02	2.19 ± 0.06	0.59 ± 0.03
MnO	0.53 ± 0.03	0.23 ± 0.01	0.53 ± 0.03
ZnO	0.32 ± 0.02	ND^d^	0.24 ± 0.02
SnO_2_	0.26 ± 0.02	0.24 ± 0.01	0.45 ± 0.03
TiO_2_	0.34 ± 0.02	ND	0.59 ± 0.03

a Dry-ash-free.

b Oxygen is calculated by difference.

c Determined by dividing the fixed-carbon content by the volatile matter content.

d Not detected.

As also shown in Table 1, the ashes from all the biomass sources contained considerably amounts of alkaline and alkaline-Earth metallic (AAEM) species (Ca, K, and Mg). It is well-known that these inorganic elements can significantly affect both the char yield and its reactivity. During the course of biomass pyrolysis, alkali elements (especially K) simultaneously catalyze the primary devolatilization reactions (for both hemicelluloses and cellulose) and the cracking and polymerization reactions of tar vapors.^30,31^ Furthermore, the presence of Ca and Mg could partly inhibit the thermal degradation of hemicelluloses.^32^ Concerning the catalytic effects of AAEM species on char combustion, potassium seems to be the most active element.^33,34^

### 3.2 Yields and Properties of Chars

Table 2 reports the yields *(y*_char_) properties determined for all the chars produced in this study, which were denoted as XX_*T*_*P*_τ (XX: feedstock type; *T*: peak temperature in °C; *P*: absolute pressure in MPa; τ: residence time of the vapor phase in s). To objectively assess the effects of pyrolysis conditions on the response variables given in Table 2, a two-level factorial design of experiments (with three replicates at the center point) was adopted for each biomass precursor. For this purpose, Minitab 17 software was used.

**Table 2:** Properties Determined for the Produced Chars^a^

char	*y*_char_^b^	*x*_FC_^c^	*x*_ash_^d^	O:C (atomic ratio)	H:C (atomic ratio)	fuel ratio^e^	HHV (MJ kg^–1^)^f^	*S*_BET_ (m^2^ g^–1^)	*V*_ultra_ (cm^3^ g^–1^)
VS_350_0.1_50	0.446	0.479	0.054	0.126	0.910	0.974	25.3	135	0.032
VS_350_0.1_150	0.427	0.450	0.078	0.082	0.840	0.889	25.6	134	0.035
VS_350_0.5_50	0.400	0.423	0.064	0.120	0.944	0.783	25.5	116	0.022
VS_350_0.5_150	0.401	0.420	0.060	0.103	0.907	0.772	24.9	127	0.029
VS_425_0.3_100^g^	0.327	0.553	0.091	0.052	0.680	1.362	26.1	164	0.046
VS_500_0.1_50	0.309	0.621	0.067	0.027	0.555	1.756	27.1	209	0.066
VS_500_0.1_150	0.342	0.624	0.064	0.038	0.572	1.776	27.5	208	0.064
VS_500_0.5_50	0.296	0.612	0.068	0.030	0.504	1.905	27.6	219	0.075
VS_500_0.5_150	0.332	0.602	0.067	0.027	0.526	1.625	27.4	217	0.069
WS_350_0.1_150	0.337	0.622	0.105	0.162	0.755	1.837	26.7	112	0.023
WS_350_0.5_150	0.337	0.654	0.106	0.226	0.766	2.116	27.1	95.1	0.015
WS_425_0.3_150^g^	0.282	0.743	0.138	0.154	0.594	3.353	26.8	132	0.031
WS_500_0.1_150	0.264	0.781	0.146	0.099	0.474	4.187	28.0	140	0.033
WS_500_0.5_150	0.262	0.815	0.142	0.108	0.473	5.144	27.8	160	0.043
CS_350_0.1_150	0.397	0.551	0.055	0.223	0.837	1.298	25.6	123	0.027
CS_350_0.5_150	0.374	0.559	0.045	0.210	0.766	1.328	27.6	143	0.032
CS_425_0.3_150^g^	0.334	0.665	0.093	0.132	0.655	2.188	27.3	158	0.044
CS_500_0.1_150	0.271	0.759	0.089	0.081	0.474	3.461	27.8	215	0.067
CS_500_0.5_150	0.301	0.734	0.082	0.141	0.527	3.013	27.7	211	0.062

a Denoted as XX_*T*_*P*_τ (XX: feedstock type; *T*: peak temperature in °C; *P*: absolute pressure in MPa; τ: residence time in s).

b Char yield (mass fraction in daf basis).

c Fixed-carbon content (mass fraction in daf basis).

d Ash content (mass fraction in dry basis).

e Determined by dividing the fixed-carbon content by the volatile matter content.

f Dry basis.

g Center point (reported values correspond to the averages of three replicates).

For VS-derived chars, results from the statistical analyses are summarized in Table S1, where it can be observed that the pyrolysis peak temperature significantly affected all the char properties assessed. As expected, an increase in the highest treatment temperature resulted in a decrease in *y*_char_ and an increase in the fixed-carbon content and heating value of the resulting chars, due to the higher extent of deoxygenation achieved. The rest of operating factors showed marginal or negligible effects on the response variables. Within the range of pressures analyzed here (0.1–0.5 MPa), none of the char properties assessed were significantly affected by this factor, suggesting that the previously reported increase in the fixed-carbon content with pressure^3,35^ should be restricted to more severe pressurization conditions (i.e., in the range of 0.5–1.1 MPa). With regard to the residence time of the gas phase, a marginal effect was observed for only the atomic H:C ratio (for the interaction effect *T·τ*).

For both WS- and CS-derived chars, for which the residence time was not included in the statistical study due to practical reasons (the high carrier gas flow rates that were required for experiments at the lowest gas residence time caused blockages in the outlet tubing and subsequent overpressure generation), results from the corresponding statistical analyses are presented in Tables S2 and S3, respectively. As observed in the case of VS, the highest treatment temperature was the most important factor affecting the yields and properties of produced chars for both WS and CS. However, the effect of the absolute pressure (either the main effect *P* or the interaction effect *T·P*) on the properties of produced chars was more relevant when CS was used as precursor. As can be deduced from Table S3, at the highest level of temperature (i.e., 500 °C), an increased pressure led to an increase in the atomic O:C ratio and related decreases in both the heating value and fuel ratio. This finding could be explained by a slightly increased trapping of volatiles when pyrolysis was conducted at 0.5 MPa. In fact, the mass yield obtained for the CS_500_0.5_150 char was 11.1% higher than that of CS_500_0.1_150. The observed higher oxygen content in the resulting CS-derived char (when pyrolysis pressure was set to 0.5 MPa) agrees well with earlier studies. Wafiq et al.^36^ reported an increase in the oxygen content in Miscanthus-derived chars when the pyrolysis pressure raised from 0.1 to 1.0–1.5 MPa, whereas Qin et al.^37^ recently reported a marked increase in the content of oxygenated functional groups on the surface of pine-nut-shell-derived chars when the pressure raised from 0.1 to 1.0–2.0 MPa.

The reason behind the observed more significant effect of the absolute pressure on volatile trapping for CS-derived chars, with respect to the other biomass types studied here, could possibly be attributed to the different role played by the inherent inorganic constituents. In this context, it could be assumed that the above-mentioned catalytic effect of potassium during the thermal degradation of CS was weaker than in the other two cases (VS and WS). In addition to the relatively low content of K in the CS ashes (9.85 wt % as K_2_O, as shown in Table 1), the availability of active K-containing species during the course of pyrolysis could also be limited.

### 3.3 Combustion Behavior of Chars

**Figure 1:**
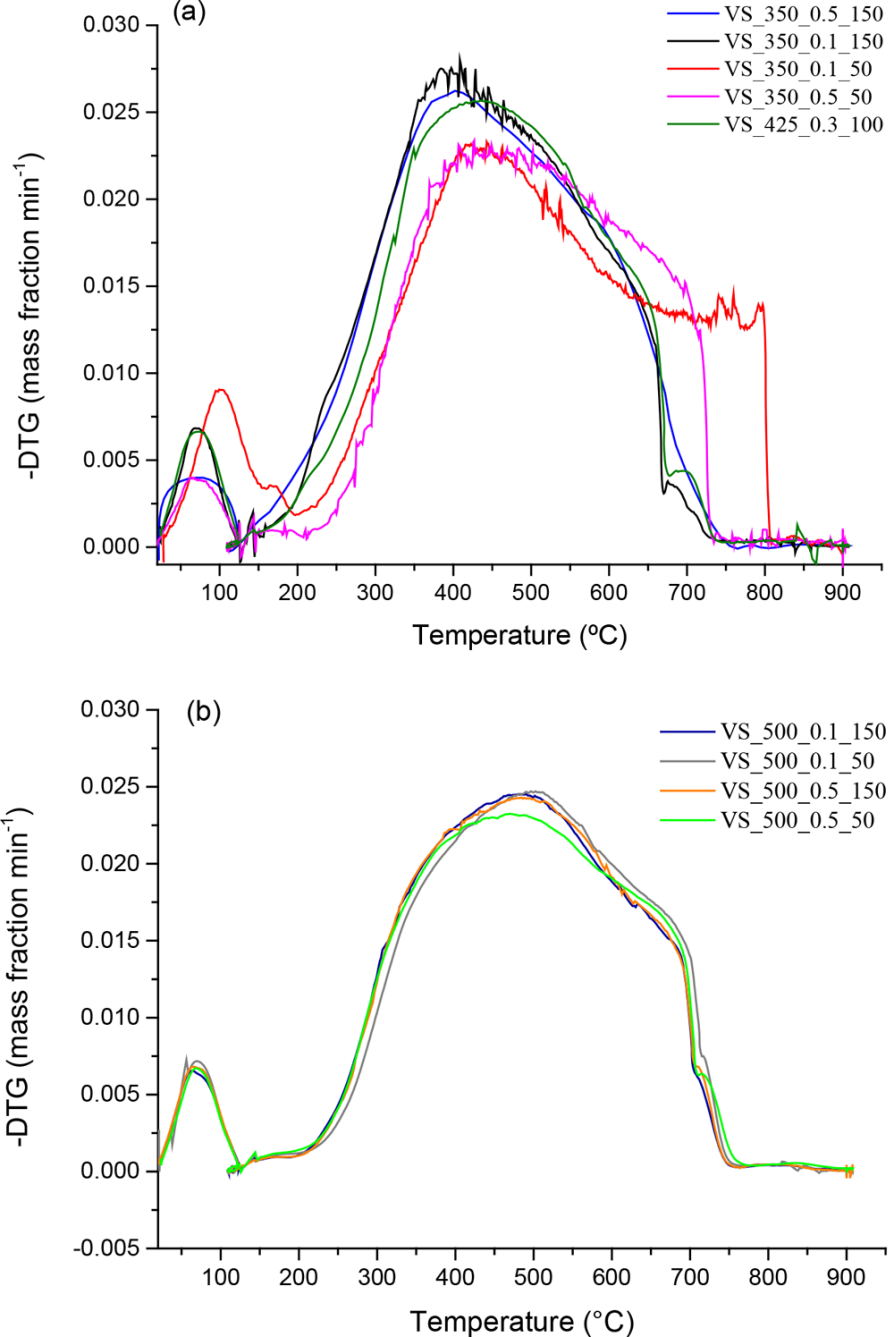
Differential thermogravimetric (DTG) combustion profiles of VS-derived chars: (a) chars produced at 350 and 425 °C; (b) chars produced at 500 °C.

**Figure 2:**
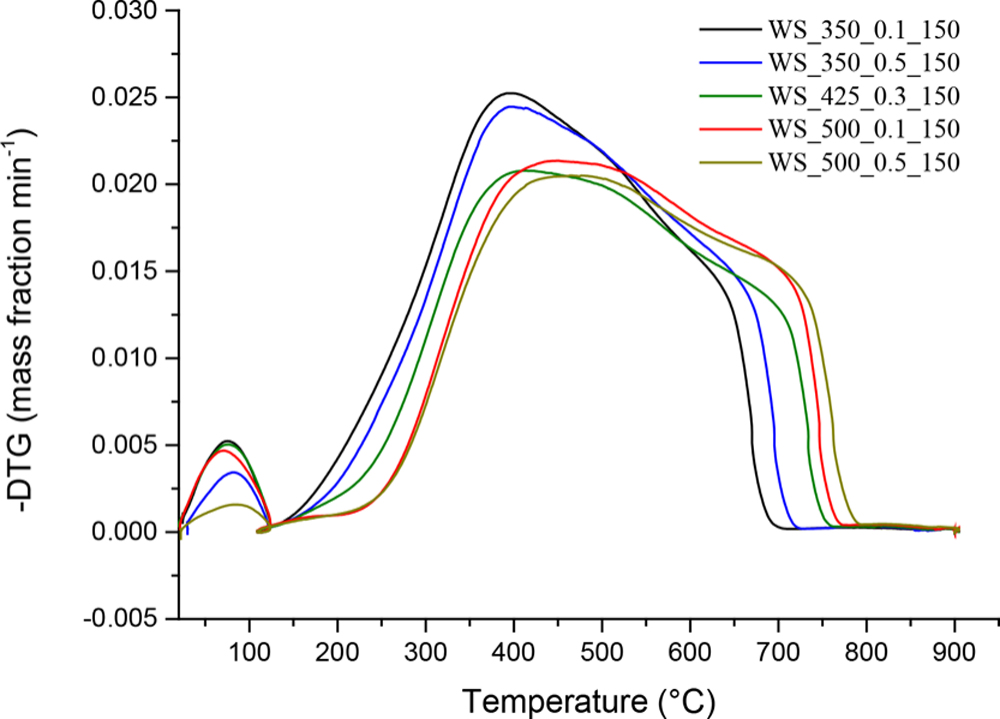
DTG combustion profiles of WS-derived chars.

**Figure 3:**
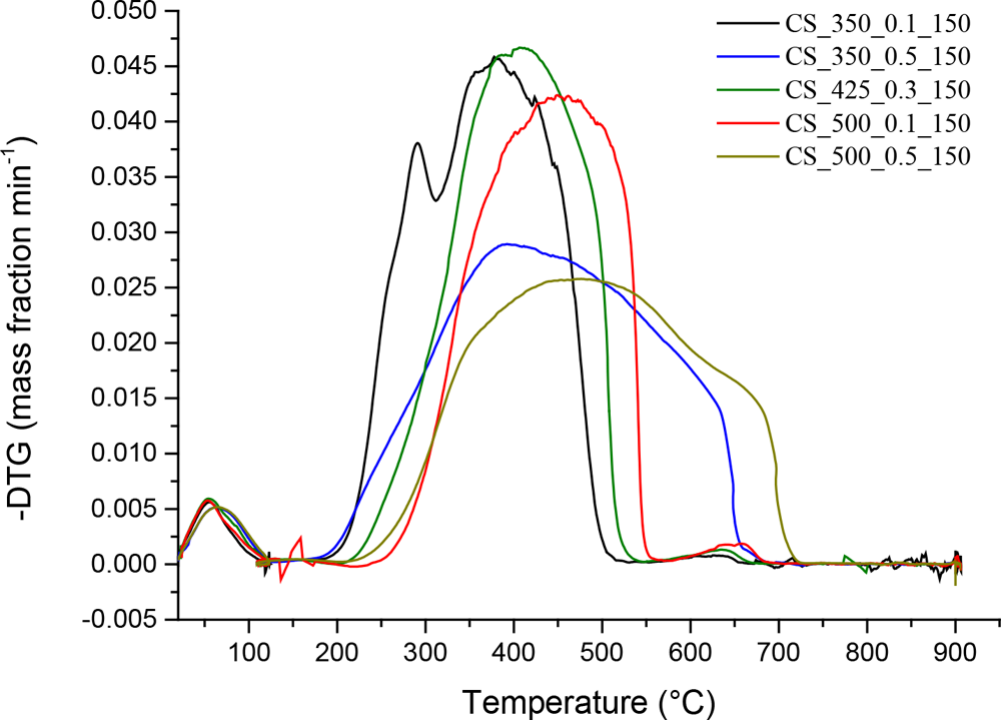
DTG combustion profiles of CS-derived chars.

Figures 1–3 show the DTG combustion profiles for VS-, WS-, and CS-derived chars. An example of how the ignition and burnout temperatures were estimated from the TG/DTG combustion profile is given in Figure S2. Combustion of biomass-derived chars usually takes place according to a multistep process, during which at least two distinct DTG peaks (those corresponding to solid devolatilization and char oxidation) can easily be distinguished.^38^ Nevertheless, these two DTG peaks were clearly observed for only one char (CS_350_0.1_150, as shown in Figure 3). For the rest of chars produced in the present study, the DTG curves only exhibited a main mass-loss peak. At temperatures below *T*_max_, this peak could mainly be due to the decomposition of volatiles that remained in the carbonized solid (as well as remaining fractions of hemicelluloses and cellulose, especially for chars pyrolyzed at 350 °C), while at temperatures above *T*_max_, it could be ascribed to the reaction of more condensed structures.^11^ The relative abundance of more stable forms of carbons may be related to the configuration of the pyrolysis reactor (in which the carrier gas did not pass through the bed), which might result in a higher carbonization efficiency due to the extended contact time between the primary volatiles and the solid matrix.

**Figure 4:**
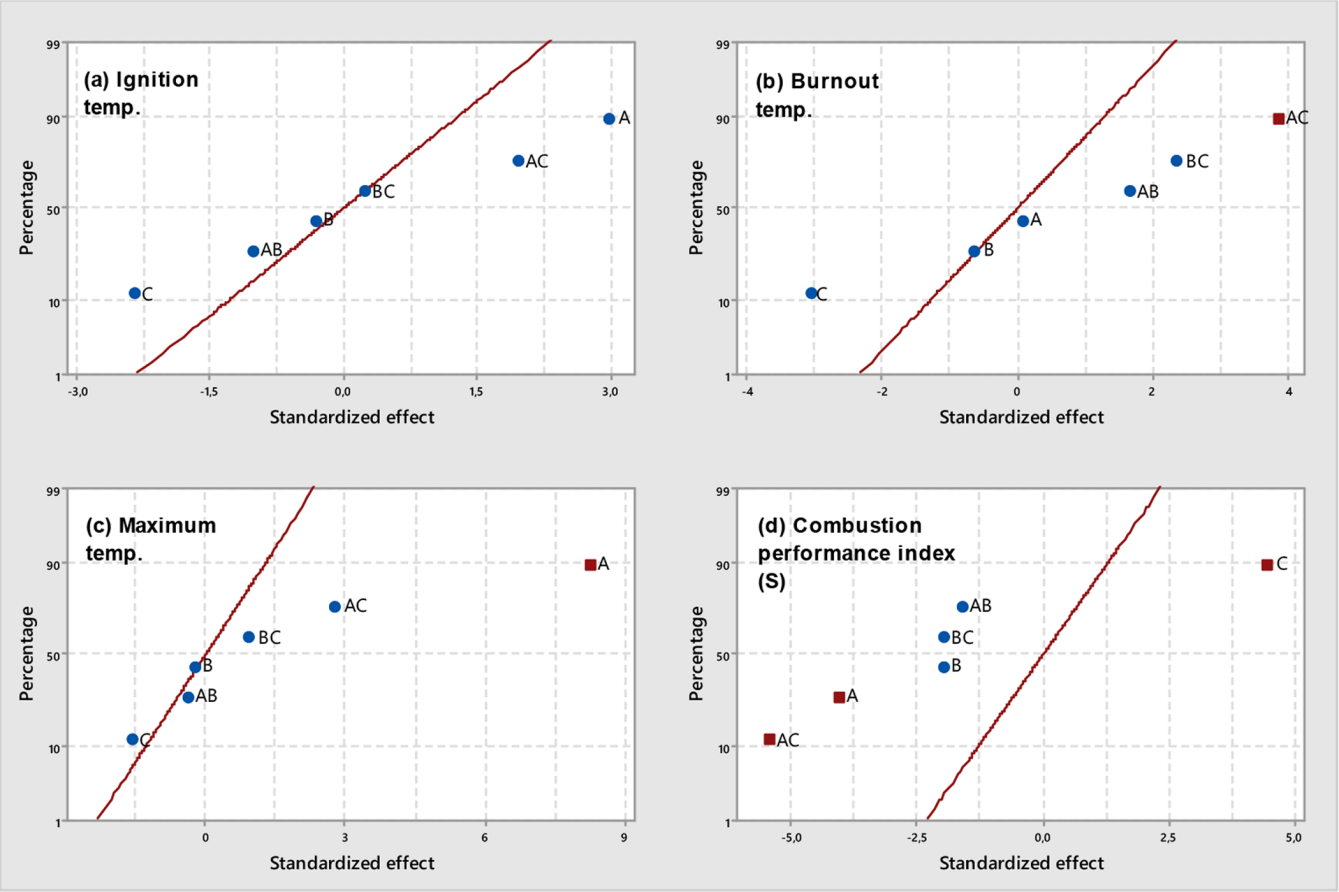
Normal plots of standardized effects (α = 0.05) for VS-derived chars: (a) *T*_i_, (b) *T*_b_, (c) *T*_max_, and (d) *S* (square, significant effect; circle, not significant effect; *A*, temperature; *B*, pressure; *C*, residence time).

Table 3 lists the characteristic temperatures and combustion performance indices, which were calculated according to the methodology described in Section 2.3. For VS-derived chars, results from the statistical analyses of the data given in Table 3 revealed a significant effect of the gas residence time, pyrolysis peak temperature, and interaction between them on the combustion performance index (see the normal plot of standardized effects in Figure 4d and the summary statistics in Table S4). At low pyrolysis peak temperatures, an increase in the gas residence time led to higher values of *S*, whereas a marked decrease in the combustion performance index was ascribed to higher values of both *T* and τ factors. These relatively low values of *S* could mainly be explained by the related increase in the burnout temperature when both the pyrolysis peak temperature and gas residence time were set at their highest levels (see Figure 4b). The important role that the gas residence time seems to play in the combustion performance was somewhat unexpected in view of the almost negligible effects of τ on the measured properties of VS-derived chars. It would be expected that an increase in the residence time of the gas phase should result in a higher carbonization efficiency, since the primary volatiles have more time to undergo secondary charring reactions, thus increasing the fixed-carbon content, which is often related to higher values of *T*_b_. Nevertheless, the fixed-carbon content of VS-derived chars was only significantly affected by the peak pyrolysis temperature (see Table S1), suggesting that the residence time of the gas phase could influence other features of the resulting chars related to, for instance, their chemical and/or morphological structure. Further studies would be needed to clarify the role of the gas residence time in the enhancement (or decrement) of char reactivity.

**Table 3:** Combustion Patterns Determined for the Produced Chars^a^

char	*T*_i_ (°C)	*T*_b_ (°C)	*T*_max_ (°C)	*S* · 10^7^ (wt %^2^ min^–2^ °C^3–^)
VS_350_0.1_50	297	788	424	0.555
VS_350_0.1_150	278	661	386	1.137
VS_350_0.5_50	310	720	431	0.621
VS_350_0.5_150	274	671	382	1.036
VS_425_0.3_100^b^	296	678	430	0.910
VS_500_0.1_50	320	710	497	0.692
VS_500_0.1_150	306	699	485	0.758
VS_500_0.5_50	300	702	467	0.626
VS_500_0.5_150	306	700	486	0.644
WS_350_0.1_150	275	660	397	0.895
WS_350_0.5_150	284	684	399	0.860
WS_425_0.3_150^b^	299	712	416	0.640
WS_500_0.1_150	316	738	446	0.525
WS_500_0.5_150	317	750	447	0.472
CS_350_0.1_150	278	485	381	4.566
CS_350_0.5_150	281	643	392	1.321
CS_425_0.3_150^b^	310	515	416	3.179
CS_500_0.1_150	333	542	449	2.560
CS_500_0.5_150	315	694	475	0.798

a Denoted as XX_*T*_*P*_τ (XX: feedstock type; *T*: peak temperature in °C; *P*: absolute pressure in MPa; τ: residence time in s).

b Center point (reported values were calculated from the average data of the three replicates).

**Figure 5:**
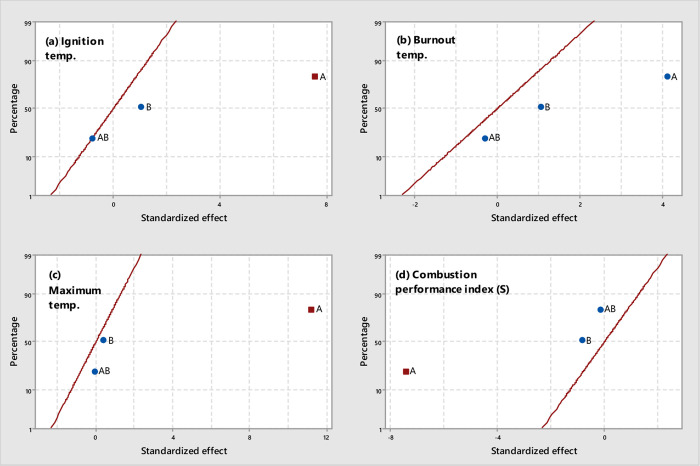
Normal plots of standardized effects (α = 0.05) for WS-derived chars: (a) *T*_i_, (b) *T*_b_, (c) *T*_max_, and (d) *S* (square, significant effect; circle, not significant effect; *A*, temperature; *B*, pressure).

**Figure 6:**
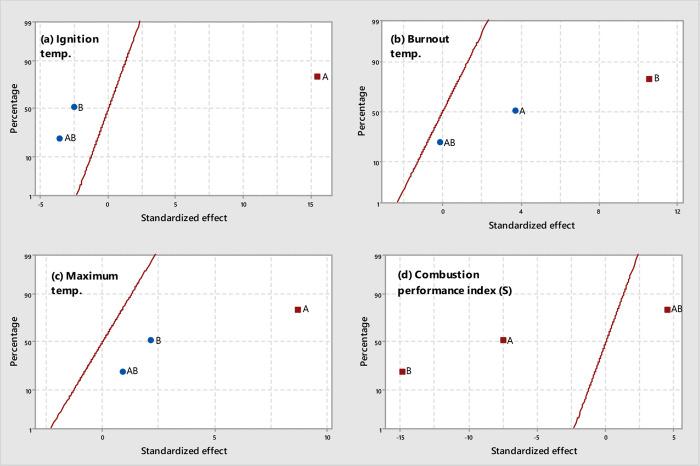
Normal plots of standardized effects (α = 0.05) for CS-derived chars: (a) *T*_i_, (b) *T*_b_, (c) *T*_max_, and (d) *S* (square, significant effect; circle, not significant effect; *A*, temperature; *B*, pressure).

The influence of pyrolysis pressure and peak temperature on the combustion performance of both WS- and CS-derived chars is summarized graphically in Figures 5 and 6, respectively (the results from statistical analyses are given in Tables S5 and S6, respectively). For chars produced from wheat straw, it can be seen that the pyrolysis peak temperature was the only factor that negatively affected the combustion performance, leading to a marked increase in both *T*_i_ and *T*_ma__*x*_ values and a related significant decrease in the value of *S* when chars were produced at the highest peak temperature. Contrary to what was observed for chars produced from VS and WS, the combustion performance of CS-derived chars was strongly affected by pyrolysis pressure. As shown in Figure 6d (and reported in Table A.6), the pressure applied during pyrolysis exerted a more pronounced effect than peak temperature on the combustion performance index values of resulting chars. The poorer combustion performance observed for CS-derived chars produced at the highest level of pressure, despite their relatively higher oxygen content, agrees with the previous results reported by Recari et al.^16^ for wood spruce chars and could be related to differences in the oxygen diffusion rate at relatively high temperatures, where the combustion is under both kinetic and internal diffusion control.^39,40^ Unfortunately, the textural properties reported in Table 2 (*S*_BET_ and *V*_ultra_) did not show any significant influence of pressure. This finding suggests that more advanced textural characterization techniques—rather than traditional N_2_ and CO_2_ adsorption isotherms—are required to better explore the wide microporosity and mesopororisity domains in order to find relevant differences that could affect the oxygen diffusion rate.

The large variability in the combustion-related variables among the chars produced from different biomass precursors could suggest that the effect of the feedstock on the combustion behavior was much stronger than those of the pyrolysis conditions. The highest value of *S* was measured for the CS_350_0.1_150 char (4.566 × 10^–7^ wt %^2^ min^–2^ °C^3–^), which was much higher than the highest *S* values measured for both VS- and WS-based chars (1.137 × 10^–7^ and 0.895 × 10^–7^ wt %^2^ min^–2^ °C^3–^, respectively).

### 3.4 Apparent Kinetic Parameters and Char Reactivity

The estimation of the apparent kinetic parameters (*E*_a_ and *A*) was performed according to the CR procedure (see Section 2.4) for a conversion range of 0.1 ≤ α ≤ 0.9. As a preliminary step, the resulting CR plots obtained for a number of expressions of *g*(α) (those corresponding to different reaction mechanisms, as shown in Table S7) were compared for a given char (VS_500_0.5_150). The best linear fit to the CR plot was observed for the F3/2 chemical reaction mechanism (see Figure S3). Since the aim of this study was to compare the relative reactivity of biomass-derived chars, *E*_a_ and *A* were estimated for all of them by assuming the same kinetic expression (F3/2).

The calculated kinetic parameters are summarized in Table 4, which also lists the average values of the temperatures range (*T*_avg_) and the coefficients of determination (*R*^2^) obtained for the linear fit to the CR plots. To take into account the well-known kinetic compensation effect, the relative reactivity (*R*) with respect to a reference case was calculated according to the following equation^25^
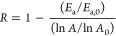
7where *E*_a,0_ and *A*_0_ correspond to the kinetic parameters for the reference case. A negative sign of *R* indicates a lower reactivity than that of the reference case. In Table 4, two relative reactivity values are reported: *R*_i_ and *R*_j_. The first one was calculated with respect to the most reactive char for the same biomass feedstock, whereas *R*_j_ was calculated using the most reactive char evaluated in the present study (CS_350_0.1_150) as the reference case.

**Table 4:** Estimated Apparent Kinetic Parameters from the CR Plot and Relative Reactivities

char	*E*_a_ (kJ mol^–1^)	*A* (min^–1^)	*R*^2^	*T*_avg_ (°C)	*R*_i_ (−)	*R*_j_ (−)
VS_350_0.1_50	29.16	7.739	0.9930	534	–0.437	–1.17
VS_350_0.1_150	34.40	32.10	0.9970	458	0	–0.512
VS_350_0.5_50	36.67	29.22	0.9944	508	–0.0957	–0.657
VS_350_0.5_150	33.43	26.01	0.9958	463	–0.0345	–0.565
VS_425_0.3_100^a^	36.74	38.17	0.9968	481	–0.0172	–0.539
VS_500_0.1_50	36.74	30.23	0.9968	503	–0.0868	–0.644
VS_500_0.1_150	35.80	28.53	0.9965	492	–0.0773	–0.629
VS_500_0.5_50	34.29	21.27	0.9948	497	–0.131	–0.711
VS_500_0.5_150	35.82	28.51	0.9956	493	–0.0781	–0.631
WS_350_0.1_150	34.26	31.78	0.9970	455	0	–0.511
WS_350_0.5_150	34.34	27.24	0.9962	474	–0.0522	–0.585
WS_425_0.3_150^a^	34.97	24.40	0.9947	498	–0.112	–0.670
WS_500_0.1_150	41.84	58.88	0.9834	473	–0.0156	–0.566
WS_500_0.5_150	40.99	47.15	0.9830	480	–0.0545	–0.622
CS_350_0.1_150	51.97	2770	0.9942	367	0	0
CS_350_0.5_150	37.50	60.45	0.9966	447	–0.428	–0.428
CS_425_0.3_150^a^	60.77	7114	0.9969	406	–0.0422	–0.0422
CS_500_0.1_150	65.72	11 223	0.9953	433	–0.0713	–0.0713
CS_500_0.5_150	40.41	62.76	0.9966	493	–0.526	–0.526

a Center point (reported values were calculated from the average data of the three replicates).

**Figure 7:**
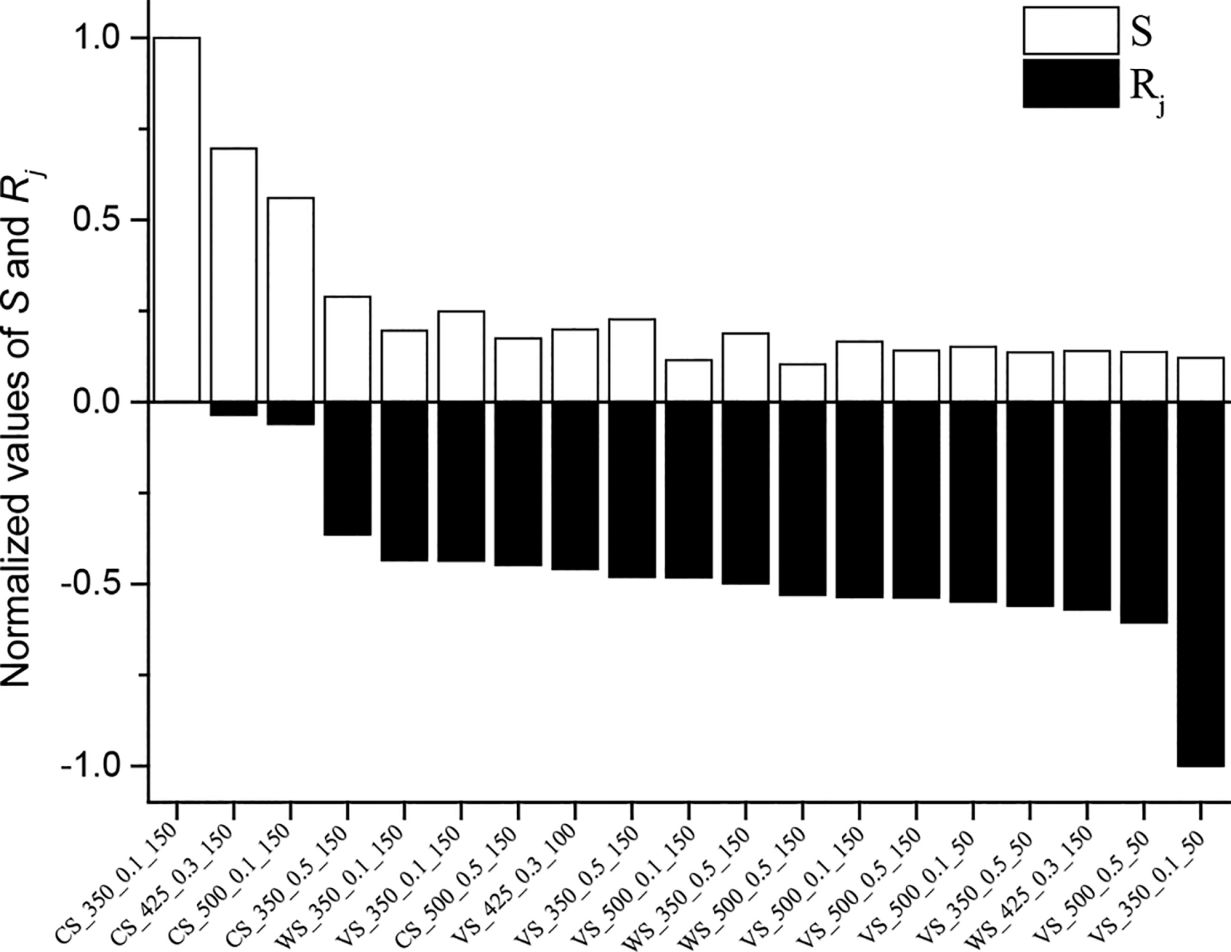
Comparison between the normalized values of *S* and *R*_j_.

The values of *R*_j_ reported in Table 4 were in acceptable agreement with the values of *S* listed in Table 3 (Spearman’s rank correlation coefficient of 0.8404 with a *p*-value of 6.69 × 10^–6^). To better reflect the level of association between *R*_j_ and *S*, Figure 7 shows, for each char, the normalized values of both indices. The reasonable level of similarity between the combustion performance index and relative reactivity suggests that *S* can be used as a convenient (and fast) rough indicator of the combustion reactivity of biomass-derived chars.

The CS-derived chars also exhibited the largest variability in the values of the apparent kinetic parameters. For the most reactive CS-derived chars, notably higher values for both *E*_a_ and *A* were found. For their part, the apparent kinetic parameters for the least reactive CS-derived chars were more similar in magnitude to those estimated for both VS- and WS-derived chars.

### 3.5 Multivariate Analysis

To further explore possible relationships that can be helpful to explain the different combustion patterns, partial least-squares (PLS) regression was performed using the “pls” package for the R environment.^41^ PLS, which is a linear multivariate method for relating independent variables with responses, is often helpful when numerous highly correlated predictor variables are present.^42^ The approach is based on defining a relatively few latent variables (i.e., components) as linear combinations of the original independent variables that can then predict the responses. The influence of a given independent variable on a given response can be assessed using the variable importance in projection (VIP) scores, which reflects the relative importance of each independent variable on the response.^43^

The dependent variables (*X*) selected for PLS were the hemicelluloses, cellulose, lignin, potassium, and calcium contents (in wt %) in the biomass feedstock (Hemicel, Cel, Lignin, K-bio, and Ca-bio); the fixed-carbon content (*x*_FC_, in mass fraction in daf basis), the atomic O/C and H/C ratios, the specific surface area (*S*_BET_, in m^2^ g^–1^) and HHV values (in kJ kg^–1^) measured for chars (and also listed in Table 2); and the pyrolysis operating conditions (peak temperature and absolute pressure; *T* and *P*). Residence time of the gas phase (τ) was not considered, because its effect was only assessed for VS-derived chars. The combustion performance index (*S*) was selected as a response variable. Cross-validation using 10 random segments was conducted to choose the number of components that minimized the root-mean-square error of prediction (RMSEP).

**Figure 8:**
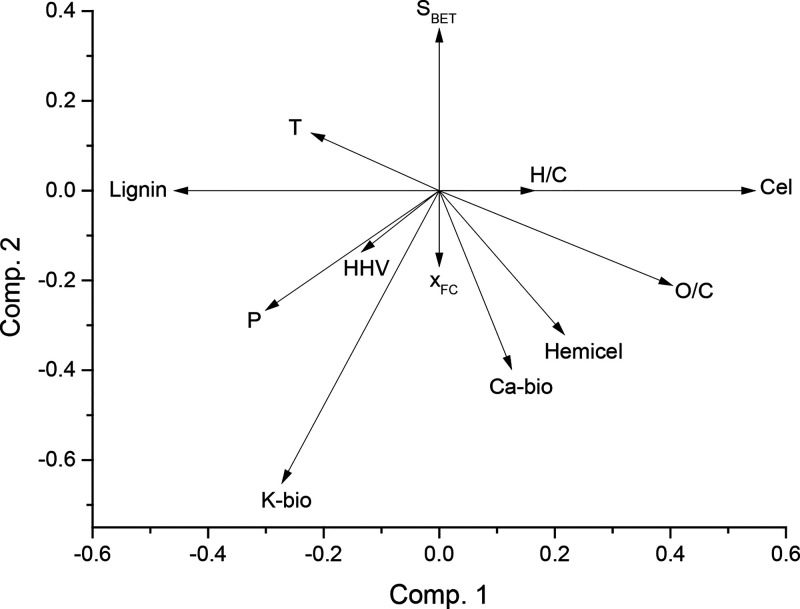
PLS loading-weights plot for dependent variables.

Results from PLS regression with three components revealed that 35.7 and 27.7% of the total variance observed in *S* was explained by component 1 and component 2, respectively (see the Supporting Information for full results). From the PLS loading-weights plot shown in Figure 8, it can be seen that none of the independent variables were positively correlated with both the first and second components In addition, K-bio and, to a lesser extent, HHV and P were the strongest negative variables affecting *S*. The negative effect of potassium on the combustion performance index could mainly be explained by differences in the pyrolysis behavior. A relatively high content of K in the biomass feedstock could result in a greater extent of secondary reactions (both cracking and coking), leading to the formation of more stable chars. In fact, the pyrolysis of WS, which had the highest potassium content, led to chars with higher fixed-carbon contents compared to those produced from VS and CS at the same operating conditions (see Table 2) and despite the relatively low content of lignin in WS.

On the other hand, Ca-bio was positively correlated with the first component and negatively correlated with the second component. Given the percentages of variance explained by the first two components and the loading weights obtained for Ca-bio (0.125 and −0.397 for components 1 and 2, respectively), a globally negative effect of calcium on *S* can be deduced. However, this negative effect was much lower than that of potassium. It is generally agreed that calcium has a lower catalytic activity on the biomass pyrolysis than that of potassium, especially at temperatures below 400 °C.^44^ Although the catalytic activity on the char oxidation process of potassium is greater than that of calcium—see, for instance, the study by Abián et al.^33^—the low intrinsic reactivity of the more stable chars produced from K-rich biomass sources could act as a bottleneck and hinder the catalytic activity of inherent potassium.

**Figure 9:**
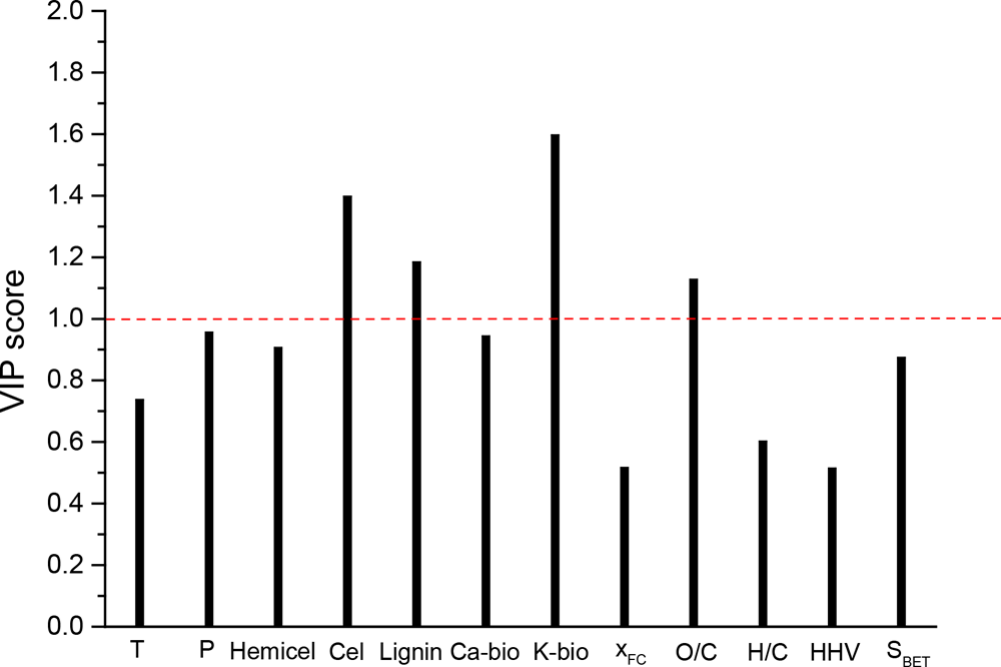
Variable importance projection (VIP) scores from PLS regression.

Figure 9 displays the VIP-scores plot for PLS model. It is widely accepted that variables having a VIP score higher than 1 can be considered as the most influential ones.^45^ Thus, and as can be seen in Figure 9, the most important dependent variables were—in addition to K-bio—Cel, Lignin, and O/C.

The negative effect of Lignin on the combustion performance of chars was confirmed (i.e., Lignin was negatively correlated with the first component). From Figure 8, it can also be seen that Cel was highly (and positively) correlated with the first component. The positive effect of Cel on the combustion performance index seems to be in disagreement with the results by Pang et al.,^15^ who reported a decrease in combustion reactivity for chars produced from some cellulose-rich biomass sources. Nevertheless, Ma et al.^46^ observed that biomass sources having relatively low cellulose to lignin content ratios exhibited poorer combustion performances. In accordance with the argument made by Yan et al.,^17^ a relatively low content of lignin could result in a lower insulation of cellulose, which could then easily decompose and lead to chars with enhanced ignition characteristics. In any case, trying to predict both the pyrolysis behavior and combustion patterns as a function of the initial contents of cellulose and lignin is extremely difficult, since the encapsulated vapor–solid interactions between biomass constituents are extremely complex. These interactions could result in significant differences in porosity development, morphology, chemical structure, and availability of oxygen-containing functional groups between synthetic component mixtures (of hemicelluloses, cellulose, and lignin) and real biomass samples, as has recently observed by Hu et al.^47^

With regard to the importance accounted for O/C, which globally favored the combustion performance (see Figure 8), it is widely believed that higher oxygen contents in char can be related to a greater availability of active sites and, therefore, a higher reactivity.^14^ As previously discussed in Section 3.2, an increased pyrolysis temperature resulted in a significant decrease in the atomic O/C ratio of resulting chars for all biomass sources. In the case of CS (the feedstock with the highest oxygen content), the substantial improvement of the O/C ratio for chars produced at 0.5 MPa and 500 °C (with respect to those produced at 0.1 MPa and 500 °C) did not translate to a better combustion performance. This finding suggests that the positive effect of O/C on *S* could be restricted to chars having less stable forms of carbon (i.e., chars produced at the lowest levels of both *T* and *P*).

## 4 Conclusions

The combustion patterns of chars were more influenced by the type of feedstock than by the pyrolysis operating conditions (for the parameters and their ranges studied here). Among the three biomass sources, corn stover appeared to be the most interesting one in order to produce highly reactive chars. Furthermore, less reactive CS-derived chars (which can be preferred for certain applications) can also be produced by increasing either the pressure or the peak temperature during the pyrolysis process. PLS regression can serve as a useful tool to evaluate the effect and importance of each explanatory variable on the combustion reactivity of biomass chars. For the feedstocks and pyrolysis process parameters assessed here, PLS regression revealed that the most important factors affecting char reactivity were the contents of K (negative effect) and cellulose (positive effect) in the raw biomass. Further studies for a wider range of biomass sources appear to be necessary to confirm the preliminary results reported here as well as confirm the usefulness of this multivariate statistical tool.

## Supporting Information Available

Tables S1–S3 (summary statistics for the regression models based on the data given in Table 2); Figure S1 (schematic diagram of the experimental pyrolysis setup), Figure S2 (an example of estimation of *T*_i_ and *T*_b_); Tables S4–S6 (summary statistics for the regression models based on the data given in Table 3); Table S7 (expressions for kinetic models); Figure S3 (CR plots for the VS_500_0.5_150 char); full results from PLS regression approach (PDF)
