# Supplementary material for: Assessing the Importance of Pyrolysis Process Conditions and Feedstock Type on the Combustion Performance of Agricultural-Residue-Derived Chars
Source: Energy Fuels. 2021 Feb 3;35(4):3174–85. doi: 10.1021/acs.energyfuels.0c04180 (PMC9161721; doi:10.1021/acs.energyfuels.0c04180)
Supplement: Supplementary file 1 — ef0c04180_si_001.pdf [file ef0c04180_si_001.pdf]

Supporting Information

Assessing the importance of pyrolysis process conditions and feedstock type on the combustion performance of agricultural residues-derived chars

Joan J. Manyà[*,†], *Darío Alvira*[†], *María Videgain*[†], *Gozde Duman*[‡], *and Jale Yanik*[‡]

[†] Aragón Institute of Engineering Research (I3A), Thermochemical Processes Group, University of Zaragoza, Escuela Politécnica Superior, crta. Cuarte s/n, 22071 Huesca, Spain

[‡] Faculty of Science, Department of Chemistry, Ege University, 35100 Bornova, Izmir, Turkey

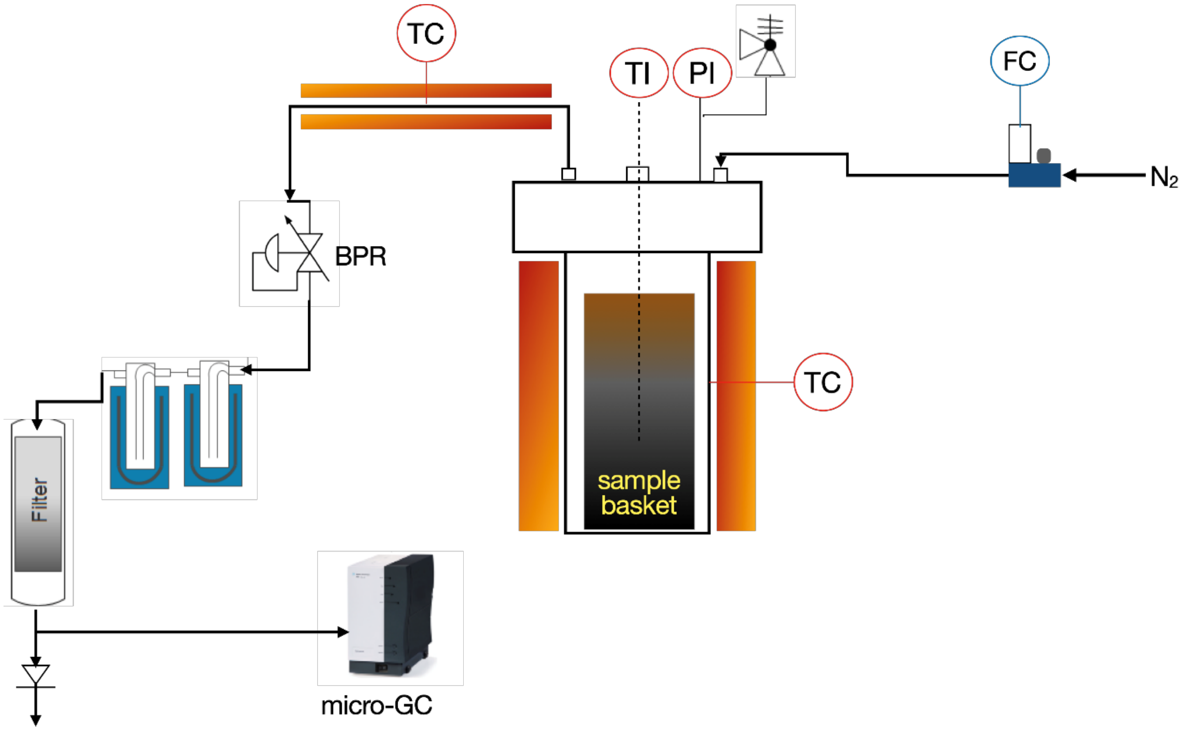

Figure S1. Schematic diagram of the experimental pyrolysis setup used in the present study.

Table S1. Summary statistics for the regression models based on the data given in Table 2 for VS-derived chars (values in brackets correspond to the *p*-values resulting from *t*-tests; significant terms are marked in bold)

Term[a]	*Response variables*							
	y_{char}	x_{FC}	*H:C ratio*	*O:C ratio*	*Fuel ratio*	*HHV*	S_{BET}	V_{ultra}
β_0	0.365	0.534	0.728	0.065	1.343	26.37	170	0.049
β_1 *(T)*	**–0.046 (0.031)**	**0.085 (0.003)**	**–0.344 (0.000)**	**–0.040 (0.015)**	**0.464 (0.002)**	**1.076 (0.001)**	**42.1 (0.000)**	**0.020 (0.001)**
β_2 *(P)*	–0.009 (0.526)	–0.015 (0.186)	0.009 (0.318)	0.005 (0.562)	–0.030 (0.538)	0.026 (0.734)	–1.42 (0.605)	0.000 (0.986)
β_3 *(τ)*	0.010 (0.476)	–0.006 (0.575)	0.000 (0.988)	–0.002 (0.793)	–0.036 (0.470)	0.026 (0.734)	0.33 (0.903)	0.001 (0.768)
β_{12} *(T·P)*	0.010 (0.486)	0.006 (0.547)	–0.016 (0.125)	0.001 (0.867)	0.047 (0.364)	0.151 (0.122)	5.08 (0.132)	0.004 (0.073)
β_{13} *(T·τ)*	0.014 (0.320)	0.002 (0.811)	**0.027 (0.039)**	0.013 (0.204)	–0.012 (0.801)	0.101 (0.247)	–2.17 (0.444)	–0.002 (0.259)
β_{23} *(P·τ)*	0.006 (0.639)	0.001 (0.931)	0.013 (0.178)	0.000 (0.989)	–0.020 (0.681)	–0.149 (0.126)	0.83 (0.760)	0.000 (0.889)
$\beta_{curvature}$	–0.027 (0.397)	0.006 (0.780)	**–0.069 (0.028)**	–0.010 (0.608)	–0.067 (0.545)	–0.311 (0.147)	4.38 (0.491)	–0.002 (0.629)
Adjusted R^2	0.603	0.906	0.984	0.719	0.920	0.960	0.970	0.952

[a] The structure of the regression model (using normalized values for factors in the range from –1 to 1) for a given response variable was the following:

$$\hat{y} = \beta_0 + \beta_1 T + \beta_2 P + \beta_3 \tau + \beta_{12} T{\cdot}P + \beta_{13} T{\cdot}\tau + \beta_{23} P{\cdot}\tau + \beta_{curvature} CenterPt$$

where β_0, β_i, β_{ij} are the intercept, linear, and 2-way interaction coefficients, respectively. $\beta_{curvature}$ is the coefficient for the center point (CenterPt) term.

Table S2. Summary statistics for the regression models based on the data given in Table 2 for WS-derived chars (values in brackets correspond to the *p*-values resulting from *t*-tests; significant terms are marked in bold)

Term	*Response variables*							
	y_{char}	x_{FC}	*H:C ratio*	*O:C ratio*	*Fuel ratio*	*HHV*	S_{BET}	V_{ultra}
β_0	0.300	0.718	0.617	0.149	3.321	27.40	128	0.028
β_1 *(T)*	**–0.037 (0.007)**	**0.080 (0.003)**	**–0.144 (0.021)**	**–0.045 (0.017)**	**1.344 (0.002)**	0.500 (0.063)	**24.2 (0.020)**	**0.010 (0.017)**
β_2 *(P)*	–0.001 (0.883)	0.016 (0.066)	0.002 (0.916)	0.018 (0.092)	**0.309 (0.040)**	0.050 (0.742)	–0.23 (0.954)	0.001 (0.729)
β_{12} *(T·P)*	–0.010 (0.883)	0.001 (0.921)	–0.003 (0.899)	–0.014 (0.148)	0.170 (0.118)	–0.150 (0.374)	8.23 (0.141)	0.004 (0.070)
$\beta_{curvature}$	–0.018 (0.062)	0.025 (0.069)	–0.023 (0.546)	0.006 (0.602)	0.032 (0.772)	–0.400 (0.186)	4.23 (0.508)	0.003 (0.278)
Adjusted R^2	0.964	0.983	0.880	0.920	0.987	0.723	0.895	0.919

Table S3. Summary statistics for the regression models based on the data given in Table 2 for CS-derived chars (values in brackets correspond to the *p*-values resulting from *t*-tests; significant terms are marked in bold)

Term	*Response variables*							
	y_{char}	x_{FC}	*H:C ratio*	*O:C ratio*	*Fuel ratio*	*HHV*	S_{BET}	V_{ultra}
β_0	0.336	0.651	0.651	0.164	2.275	27.18	173	0.047
β_1 *(T)*	**–0.050 (0.010)**	**0.096 (0.001)**	**–0.150 (0.009)**	**–0.053 (0.006)**	**0.962 (0.000)**	**0.515 (0.029)**	**40.0 (0.003)**	**0.018 (0.002)**
β_2 *(P)*	0.002 (0.756)	–0.004 (0.205)	–0.004 (0.788)	0.012 (0.103)	**–0.105 (0.022)**	**0.475 (0.042)**	4.00 (0.208)	0.000 (1.000)
β_{12} *(T·P)*	0.013 (0.115)	–0.082 (0.069)	0.031 (0.169)	**0.018 (0.047)**	**–0.112 (0.017)**	**–0.525 (0.034)**	–6.00 (0.111)	–0.002 (0.102)
$\beta_{curvature}$	–0.002 (0.0838)	0.014 (0.055)	0.004 (0.875)	**–0.032 (0.037)**	–0.087 (0.067)	0.125 (0.499)	**–15.0 (0.046)**	–0.003 (0.151)
Adjusted R^2	0.946	0.997	0.946	0.973	0.998	0.930	0.984	0.986

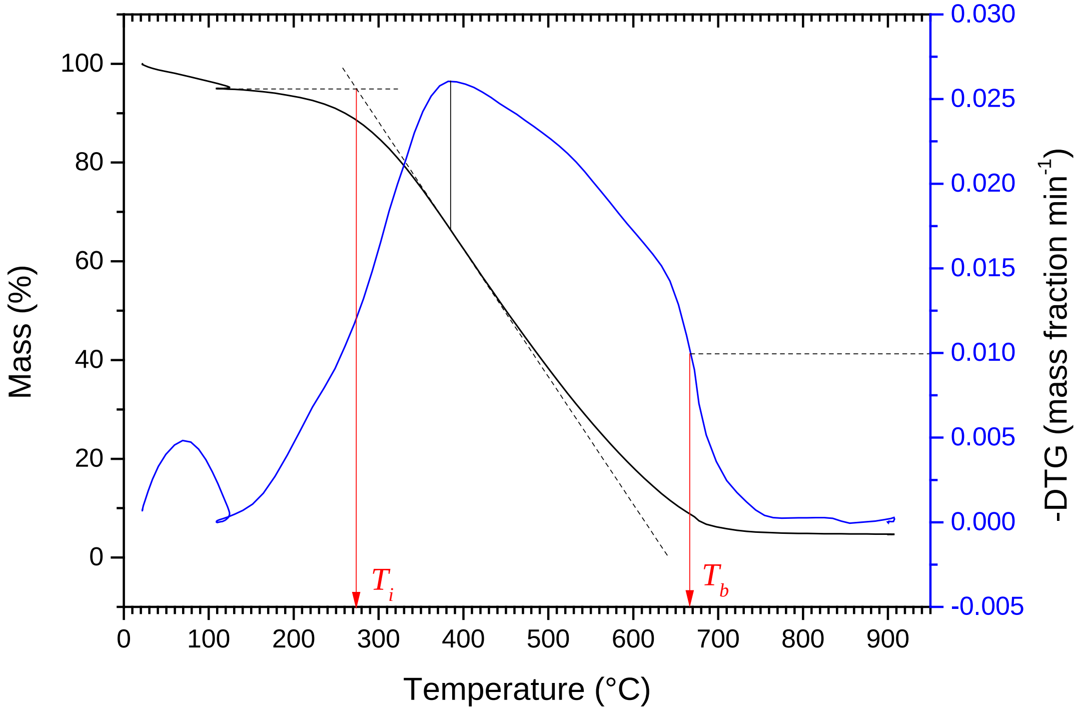

Figure S2. An example of estimation of T_i and T_b (VS_350_0.5_150 char). Black solid line: TG curve; blue solid line: DTG curve.

Table S4. Summary statistics for the regression models based on the data given in Table 3 for VS-derived chars (values in brackets correspond to the *p*-values resulting from *t*-tests; significant terms are marked in bold)

Term	*Response variables*			
	T_i	T_b	T_{max}	$S \cdot 10^{-7}$
β_0	298.5	707.4	445.0	0.749
β_1 (T)	9.41<br>(0.060)	0.34<br>(0.961)	**40.9**<br>**(0.004)**	**–0.102**<br>**(0.027)**
β_2 (P)	–1.09<br>(0.754)	–4.16<br>(0.560)	–1.31<br>(0.810)	–0.051<br>(0.138)
β_3 (τ)	–7.59<br>(0.097)	–19.7<br>(0.054)	–8.06<br>(0.205)	**0.112**<br>**(0.021)**
β_{12} (T·P)	–3.34<br>(0.371)	10.3<br>(0.203)	–2.06<br>(0.707)	–0.042<br>(0.195)
β_{13} (T·τ)	6.16<br>(0.149)	**24.3**<br>**(0.031)**	13.7<br>(0.071)	**–0.138**<br>**(0.012)**
β_{23} (P·τ)	0.66<br>(0.850)	14.8<br>(0.102)	4.44<br>(0.440)	–0101<br>(0.138)
$\beta_{curvature}$	–2.25<br>(0.850)	–31.7<br>(0.102)	–15.5<br>(0.265)	**0.189**<br>**(0.045)**
Adjusted R^2	0.583	0.752	0.879	0.877

Table S5. Summary statistics for the regression models based on the data given in Table 3 for WS-derived chars (values in brackets correspond to the *p*-values resulting from *t*-tests; significant terms are marked in bold)

Term	*Response variables*			
	T_i	T_b	T_{max}	$S \cdot 10^{-7}$
β_0	298.0	708.0	422.2	0.688
β_1 (T)	**18.5**<br>**(0.017)**	36.0<br>(0.055)	**24.2**<br>**(0.008)**	**–0.190**<br>**(0.018)**
β_2 (P)	2.50<br>(0.417)	9.00<br>(0.414)	0.75<br>(0.764)	–0.022<br>(0.478)
β_{12} (T·P)	–2.00<br>(0.503)	–3.00<br>(0.766)	–0.25<br>(0.919)	–0.005<br>(0.876)
$\beta_{curvature}$	–1.33<br>(0.757)	–0.30<br>(0.982)	–8.25<br>(0.131)	–0.034<br>(0.470)
Adjusted R^2	0.900	0.698	0.955	0.898

Table S6. Summary statistics for the regression models based on the data given in Table 3 for CS-derived chars (values in brackets correspond to the *p*-values resulting from *t*-tests; significant terms are marked in bold)

Term	*Response variables*			
	T_i	T_b	T_{max}	$S \cdot 10^{-7}$
β_0	301.8	591.0	424.2	2.311
β_1 (T)	**22.2 (0.004)**	27.0 (0.067)	**37.8 (0.013)**	**–0.632 (0.017)**
β_2 (P)	–3.75 (0.122)	**77.5 (0.009)**	9.25 (0.168)	**–1.252 (0.004)**
β_{12} (T·P)	–5.25 (0.068)	–1.50 (0.857)	3.75 (0.481)	**0.371 (0.047)**
$\beta_{curvature}$	8.92 (0.056)	**–187.0 (0.004)**	**84.4 (0.006)**	**0.861 (0.021)**
Adjusted R^2	0.978	0.985	0.975	0.983

Table S7. Expressions of functions *f(α)* and *g(α)* and their corresponding mechanism[1]

Code	Name	*f(α)*	*g(α)*	Mechanism
F1	first order	$1-\alpha$	$-\ln(1-\alpha)$	chemical reaction
F2	second order	$(1-\alpha)^2$	$(1-\alpha)^{-1}-1$	
F3	third order	$\frac{1}{2}(1-\alpha)^3$	$(1-\alpha)^{-2}-1$	
F1/3	one-third order	$\frac{3}{2}(1-\alpha)^{1/3}$	$1-(1-\alpha)^{2/3}$	
F3/4	three-quarters order	$4(1-\alpha)^{3/4}$	$1-(1-\alpha)^{1/4}$	
F3/2	one and a half order	$2(1-\alpha)^{3/2}$	$(1-\alpha)^{-1/2}-1$	
D1	parabola law	$1/(2\alpha)$	α^2	1-D diffussion
D2	Valensi equation	$\left[-\ln(1-\alpha)\right]^{-1}$	$\alpha+(1-\alpha)\ln(1-\alpha)$	2-D diffussion
D3	Jander equation	$\frac{3}{2}(1-\alpha)^{2/3}\left[1-(1-\alpha)^{1/3}\right]^{-1}$	$\left[1-(1-\alpha)^{1/3}\right]^2$	3-D diffussion, spherical
D4	Ginstling−Brounstein equation	$\frac{3}{2}\left[(1-\alpha)^{-1/3}-1\right]^{-1}$	$1-\frac{2}{3}\alpha-(1-\alpha)^{2/3}$	3-D diffussion, cylindrical

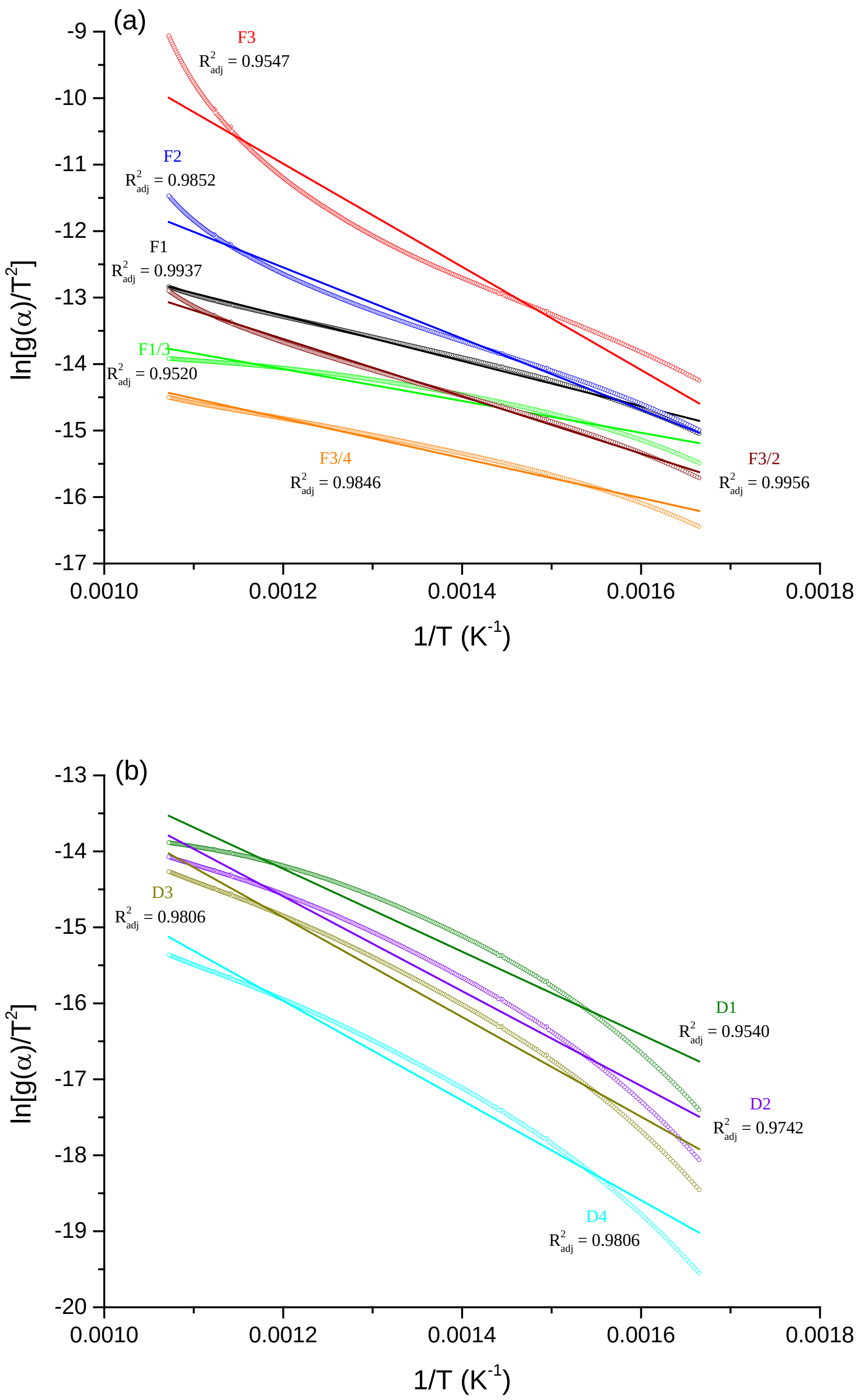

Figure S3. CR plots for the VS_500_0.5_150 char adopting different expressions of *g(α)*: (a) those corresponding to some chemical reactions mechanisms, and (b) those corresponding to some diffusion-based mechanisms.

Results from PLS regression approach (response: *S*)

a) Cross validation (number of components)

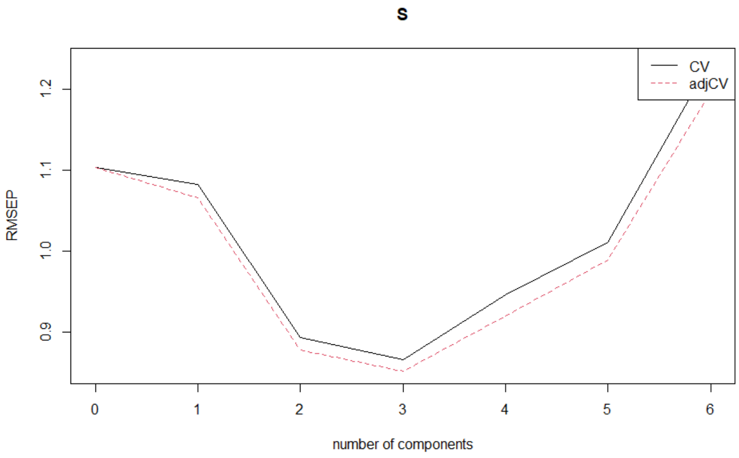

CV is the cross-validation estimate, and *adjCV* is the bias-corrected cross-validation estimate. RMSEP is the root mean square error of prediction for the response ($S \cdot 10^7$ in wt.%^{2} min^{-2} °C^{-3}).

b) Regression coefficients

T	–0.10229
P	–0.31871
Hemicel	0.01966
Cel	0.39977
Lignin	–0.29230
Ca-bio	–0.07526
K-bio	–0.45752
x_{FC}	–0.08698
O/C	0.19583
H/C	0.10221
HHV	–0.14983
S_{BET}	0.08056

c) Scores

X variable	Comp. 1	Comp. 2	Comp. 3
VS_350_0.1_50	–0.0084	1.3688	–2.3386
VS_350_0.1_150	–0.3888	1.2350	–2.1015
VS_350_0.5_50	–0.6190	0.2496	–3.3498
VS_350_0.5_150	–0.7072	0.4294	–3.1863
VS_425_0.3_100	–1.4508	0.4745	–0.7962
VS_500_0.1_50	–1.9051	0.8189	1.1659
VS_500_0.1_150	–1.8710	0.7370	1.1518
VS_500_0.5_50	–2.6619	–0.2776	0.8508
VS_500_0.5_150	–2.6250	–0.2203	0.7216
WS_350_0.1_150	1.7279	–1.1763	–1.1110
WS_350_0.5_150	1.4861	–2.3811	–1.9305
WS_425_0.3_150	0.8776	–1.9094	0.0274
WS_500_0.1_150	0.3038	–1.8636	1.5758
WS_500_0.5_150	–0.3030	–2.7549	1.2325
CS_350_0.1_150	3.1297	2.0209	–0.1756
CS_350_0.5_150	2.0217	0.5770	–0.0340
CS_425_0.3_150[f]	1.4832	0.8944	1.5685
CS_500_0.1_150	0.8425	1.3329	3.8614
CS_500_0.5_150	0.6676	0.4447	2.8678

d) Loadings and loading-weihgts

Loadings

Char	Comp. 1	Comp. 2	Comp. 3
T	–0.316	–0.127	0.417
P	–0.102	–0.261	0.000
Hemicel	0.454	–0.422	0.170
Cel	0.513	–0.114	0.251
Lignin	–0.510	0.214	–0.233
Ca-bio	0.421	–0.481	0.143
K-bio	0.213	–0.646	0.000
x_{FC}	0.000	–0.397	0.406
O/C	0.560	–0.108	–0.126
H/C	0.187	0.250	–0.446
HHV	0.000	–0.294	0.420
S_{BET}	–0.365	0.190	0.369

Loading-weights

Char	Comp. 1	Comp. 2	Comp. 3
T	–0.221	0.128	0.448
P	–0.300	–0.266	0.000
Hemicel	0.216	–0.320	0.178
Cel	0.546	0.000	0.277
Lignin	–0.458	0.000	–0.254
Ca-bio	0.125	–0.397	0.147
K-bio	–0.272	–0.651	0.000
x_{FC}	0.000	–0.168	0.403
O/C	0.403	–0.211	–0.180
H/C	0.164	0.000	–0.494
HHV	–0.134	–0.136	0.277
S_{BET}	0.000	0.360	0.299

e) Variable importance in projection (VIP)

The VIP scores were defined as Mehmood et al.[2]:

$$VIP_j = \left(\frac{p\sum_{a=1}^{A}\left[SS_a \left(w_{aj} / \left\| w_a \right\|^2 \right) \right]}{\sum_{a=1}^{A} SS_a} \right)^{1/2} \quad \text{(S1)}$$

The basis behind this score is to assess the importance of each variable *j* being reflected by *w* (loading weigths) from each component *a*. SS_a is the sum of squares explained by the *a*th component, whereas *p* is the number of components finally selected (3 in our case). The VIP scores are shown in Figure 9.

References

(1) Chong, Y. Y.; Thangalazhy-Gopakumar, S.; Gan, S.; Ng, H. K.; Lee, L. Y.; Adhikari, S. Kinetics and Mechanisms for Copyrolysis of Palm Empty Fruit Bunch Fiber (EFBF) with Palm Oil Mill Effluent (POME) Sludge. *Energy Fuels* **2017**, *31* (8), 8217–8227.

(2) Mehmood, T.; Liland, K. H.; Snipen, L.; Sæbø, S. A Review of Variable Selection Methods in Partial Least Squares Regression. *Chemom. Intell. Lab. Syst.* **2012**, *118*, 62–69.
